# Oxygen controls on magmatism in rocky exoplanets

**DOI:** 10.1073/pnas.2110427118

**Published:** 2021-11-01

**Authors:** Yanhao Lin, Wim van Westrenen, Ho-Kwang Mao

**Affiliations:** ^a^Center for High Pressure Science and Technology Advanced Research, Beijing 100094, People’s Republic of China;; ^b^Department of Earth Sciences, Faculty of Science, Vrije Universiteit Amsterdam, 1081 HV Amsterdam, The Netherlands

**Keywords:** magmatism, oxygen fugacity, Fe-free basalt, high-temperature experiments

## Abstract

Refractory oxygen bound to cations is a key component of the interior of rocky exoplanets. Its abundance controls planetary properties including metallic core fraction, core composition, and mantle and crust mineralogy. Interior oxygen abundance, quantified with the oxygen fugacity (*f*O_2_), also determines the speciation of volatile species during planetary outgassing, affecting the composition of the atmosphere. Although melting drives planetary differentiation into core, mantle, crust, and atmosphere, the effect of *f*O_2_ on rock melting has not been studied directly to date, with prior efforts focusing on *f*O_2_-induced changes in the valence ratio of transition metals (particularly iron) in minerals and magma. Here, melting experiments were performed using a synthetic iron-free basalt at oxygen levels representing reducing (log *f*O_2_ = −11.5 and −7) and oxidizing (log *f*O_2_ = −0.7) interior conditions observed in our solar system. Results show that the liquidus of iron-free basalt at a pressure of 1 atm is lowered by 105 ± 10 °C over an 11 log *f*O_2_ units increase in oxygen abundance. This effect is comparable in size to the well-known enhanced melting of rocks by the addition of H_2_O or CO_2_. This implies that refractory oxygen abundance can directly control exoplanetary differentiation dynamics by affecting the conditions under which magmatism occurs, even in the absence of iron or volatiles. Exoplanets with a high refractory oxygen abundance exhibit more extensive and longer duration magmatic activity, leading to more efficient and more massive volcanic outgassing of more oxidized gas species than comparable exoplanets with a lower rock *f*O_2_.

## Significance

Oxygen is not only crucial for life as we know it but also forms the most abundant element in the outer layers of rocky planets in our own solar system and in exoplanetary systems orbiting other stars. Models for rocky (exo)planets suggest that on the order of 50% of all atoms in their rocky shells are oxygen atoms. Here we provide experimental evidence for a significant effect of planetary oxygen abundance on melting of rocks, showing that higher rock oxygen abundance leads to easier rock melting. This suggests that the extent and vigor of magmatism differ greatly between low-oxygen and high-oxygen exoplanets, opening an avenue to couple future observations of exoplanet atmospheres to interior compositions that cannot be directly observed.

Oxygen (∼21% in Earth’s atmosphere) is essential to life on Earth, and is the most abundant element in the rocky outer layers of Earth (1) as well as the other terrestrial planets in our solar system (2–4). Oxygen can be both refractory (bonded to cations—predominantly Si, Mg, Fe, and Ca—in minerals condensing from planetary disks orbiting young stars) and volatile (condensing in ices—for example, H_2_O, CO, CO_2_—in the colder outer disk parts) (e.g., ref. 5). The abundance and activity of refractory oxygen in a rock is described using the oxygen fugacity (*f*O_2_), commonly quantified relative to mineral redox buffers, for example, the IW (iron–wüstite) or MH (magnetite–hematite) buffer (6).

The *f*O_2_ conditions in planetary interiors affect the prevailing redox state of polyvalent cations, particularly transition metals (e.g., Fe^0^ versus Fe^2+^ versus Fe^3+^; Eu^2+^ versus Eu^3+^; Cr^2+^ versus Cr^3+^) (e.g., ref. 7). The oxygen fugacity in the rocky mantles of the terrestrial planets varies widely. Mercury’s rocky outer shell is oxygen depleted, with an *f*O_2_ around 5 log units below that of the iron–wüstite buffer (ρIW−5) (8). As a result, the iron content of Mercury’s mantle and crust is very low, with almost all Fe present at Fe^0^ in the core. In contrast, Earth’s upper mantle today is estimated to contain oxygen levels corresponding to *f*O_2_ of ∼ρIW+3 (6), and parts of the Martian mantle may exhibit *f*O_2_ up to ρIW+5 (e.g., ref. 9), yielding mantles with FeO abundances of 8 wt.% and 18 wt.%, respectively.

These current mantle *f*O_2_ values do not necessarily reflect oxygen fugacity conditions during planetary accretion. Mantle oxygen fugacities can change significantly during planetary growth, differentiation, and subsequent evolution. For example, models of accretion and metallic core segregation in Earth (e.g., refs. 10 and 11), based on the observed abundances of siderophile (iron-loving) elements in their mantle, suggest initial *f*O_2_ values during accretion that are Mercury-like, up to 8 log *f*O_2_ units lower than present-day mantle estimates. This suggests significant terrestrial mantle oxidation occurred after the main phase of accretion and core formation, perhaps resulting from late accretion of oxidizing agents such as H_2_O (11). Alternatively, in Earth, mantle self-oxidation is proposed to have occurred through the disproportionation reaction 3Fe^2+^O = Fe_2_^3+^O_3_ + Fe^0^ which can take place in the presence of the high-pressure mineral bridgmanite (e.g., ref. 12) or in a deep magma ocean setting (13). If iron metal can be sequestered to the core, this reaction can increase the redox state of the mantle. In Mars and the Moon, lower interior pressures prevented this process, although the increasing stability of Fe^3+^ over Fe^2+^ with increasing pressure (e.g., ref. 14) could still have resulted in significant vertical oxygen fugacity gradients in their magma oceans.

 Astronomical observations suggest that both the initial and present-day spreads in terrestrial planet oxygen abundances, masses, and sizes in our solar system are not unusually broad. Many rocky exoplanets have been characterized over the past decade, some with masses and radii far exceeding those of the rocky bodies in our own solar system (e.g., ref. 15). Such planets likely include super-Earths (with compositions perhaps similar to terrestrial planets in our solar system but with significantly higher mass and radius), but also likely include rocky planets with bulk compositions significantly different from compositions found in our solar system. Examples include rocky exoplanets enriched in elements that typically condense at very high temperatures, such as Ca and Al (16); rocky exoplanets rich in water (“ocean worlds”) (17); planets that lack a metallic core altogether (18); and planets with high or low C/O ratios (16, 19, 20). The mantle oxygen fugacity in such exoplanets could span a range similar to that found in rocky bodies in our solar system (e.g., refs. 21 and 22).

## Effects of *f*O_2_ on Exoplanet Properties

Assessing and quantifying the effects of rock oxygen fugacity variations is important, as *f*O_2_ provides fundamental controls on the first-order interior structure of rocky planets. Refractory oxygen abundance determines the proportion of iron that is reduced to the metallic form, and hence controls the core fraction of terrestrial (exo)planets (5), as well as the mineralogy (23) and viscosity (24) of planetary mantles. The oxygen fugacity of mantle rocks also plays a key role in controlling the speciation of hydrogen- and carbon-bearing species outgassing from early global magma oceans, or from erupting lava during later episodes of volcanic activity (25–28). At high *f*O_2_, volatile species such as H_2_O and CO_2_ dominate volcanic gas composition, whereas, at low *f*O_2_, the stable species are H_2_ and CO, with CH_4_ also playing a significant role. As atmospheric gas speciation strongly affects prebiotic chemosynthesis (29), rock oxygen fugacity can therefore also influence a rocky planet’s surface habitability.

## Magma Oceans on Rocky Exoplanets

Melting drives the primary differentiation of rocky planets into metallic core and silicate crust and mantle. Thermal models and measurements of the elemental and isotopic compositions of crust and mantle samples suggest that the inner solar system planets all experienced a magma ocean stage (30, 31). Molten metal and molten silicate are thought to (partially) equilibrate at the bottom of these oceans, leading to systematic depletions in siderophile element abundances in the rocky shells of Mercury, Earth, the Moon, and Mars (11, 32–34).

Equilibration between globally distributed magma and the overlying atmosphere, accompanied by significant outgassing from the convecting magma, can fundamentally alter the thickness and composition of the initial atmospheres that may have been inherited from the disk stage of a growing exoplanetary system (35, 36). The extent of melting of a rocky exoplanet during its magma ocean stage is determined by a combination of a planet’s temperature profile and the solidus (temperature below which no liquid is present) and liquidus (temperature above which only liquid is present) of the rocks constituting the planet’s outer silicate reservoir (35–37).

In current studies of rocky (exo)planet evolution, the melting behavior of planetary mantles is usually parametrized using experimental data on the melting of peridotitic or pyrolytic bulk compositions representing Earth’s mantle (38–42). As reviewed in Hirschmann (38), it has been recognized that bulk compositional differences, in terms of cation ratios such as Mg/Fe or the total abundance of alkali metals, can affect melting temperatures. Such effects have also been observed in studies of solidus temperature variations for Martian mantle compositions (43). It is similarly well established that addition of volatile compounds such as water or CO_2_ can lower the solidus of mantle compositions significantly (44, 45), but the effect of *f*O_2_ on melting of peridotitic or pyrolytic compositions is not constrained.

## High-Temperature Experiments

Studies of the effect of *f*O_2_ on magma oceans have, to date, been limited to quantifying the link between *f*O_2_ variations and changes in the redox states of the major cation iron (13, 26, 46), or linked directly to models of gas speciation in atmospheres influenced by magma ocean outgassing (35, 36, 47). Variations in solidus and liquidus temperatures of cooling magma oceans have largely been assessed in terms of variations in volatile abundances (48, 49). In this study we provide experimental evidence for a direct effect of oxygen fugacity on rock melting properties, in the absence of valence state variations in iron and in the absence of volatiles.

In order to isolate a possible effect of refractory oxygen on rock melting, we conducted a series of melting experiments on a synthetic iron-free basalt composition (50) at 1 atm and *f*O_2_ conditions (imposed by air or CO/CO_2_ gas mixtures) that cover a large section of the range of conditions found in rocky bodies in our solar system. All experiments were performed at nominally anhydrous conditions. Details of starting material synthesis, experimental setup, and conditions of our 16 experiments are given in *Methods* and in *SI Appendix*.

## Results

**Fig. 1.**
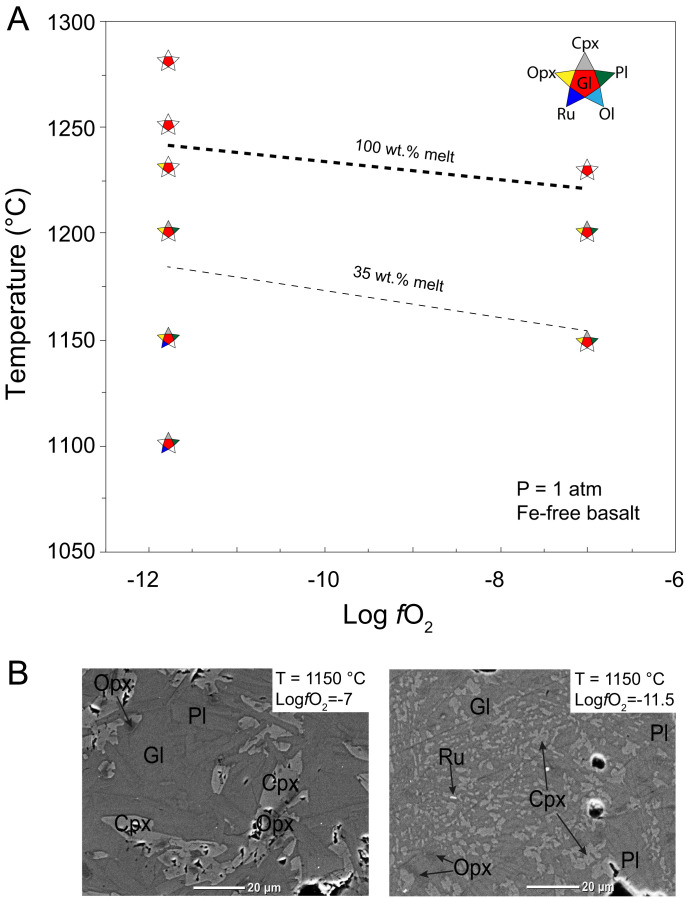
(*A*) Liquidus and phase relations of Fe-free basalt as a function of oxygen fugacity at 1 atm. The dashed curves approximate the experimental-constrained location of the liquidus and temperatures at which 35% melt is present in the system as a function of *f*O_2_. (*B*) Representative BSE images of experimental products at 1150 °C illustrating the phases present at different conditions. The experimental conditions are shown in the top right of each BSE image. Abbreviations of phases: Ol, olivine; Opx, orthopyroxene; Cpx, clinopyroxene; Pl, plagioclase; Ru, rutile; Gl, silicate glass (liquid at high-temperature conditions).

Results are summarized in Table 1 in terms of the phases present in the experiments and their proportions. Compositional data for all phases are presented in *SI Appendix*. A summary of the experimental results and backscatter electron (BSE) microscope images of representative polished run products are shown in Fig. 1. Our experiments indicate that oxygen fugacity significantly affects the liquidus temperature, the subliquidus melt percentages at a given temperature, and the appearance of mineral phases. At log*f*O_2_ = −11.5 (close to the iron–wüstite buffer) and 1,100 °C, the composition contains 13 wt.% melt, coexisting with a mineral assemblage of clinopyroxene, plagioclase, and rutile. The melt percentage increases to 18 wt.% at 1,150 °C, and the phase assemblage becomes orthopyroxene + clinopyroxene + plagioclase + rutile + melt. Rutile is not present any more at 1,200 °C when the melt percentage has increased to 36 wt.%, and clinopyroxene and plagioclase melt out between 1,200 °C and 1,230 °C (when the melt percentage reaches 99 wt.%). Orthopyroxene disappears as the last phase between 1,230 and 1,250 °C, which constrains the liquidus temperature to 1,240 ± 10 °C.

**Table 1.** Summary of experimental results

Exp.	T (°C)	Duration (h)	log*f*O_2_	Glass proportions (wt.%)	Mineral proportions (wt. %)				
					Ol	Opx	Cpx	Pl	Ru
FefreeIW6	1,280	24	CO–CO_2_ (log*f*O_2_ = −11.5)	100					
FefreeIW5	1,250	24	CO–CO_2_ (log*f*O_2_ = −11.5)	100					
FefreeIW7	1,230	40	CO–CO_2_ (log*f*O_2_ = −11.5)	99		1			
FefreeIW4	1,200	36	CO–CO_2_ (log*f*O_2_ = −11.5)	36		8	16	40	
FefreeIW2	1,150	36	CO–CO_2_ (log*f*O_2_ = −11.5)	18		8	27	46.5	0.5
FefreeIW1	1,100	36	CO–CO_2_ (log*f*O_2_ = −11.5)	13			30	56	1
Fefree-7_3	1,230	40	CO–CO_2_ (log*f*O_2_ = −7)	100					
Fefree-7_1	1,200	36	CO–CO_2_ (log*f*O_2_ = −7)	58		6	15	21	
Fefree-7_2	1,150	36	CO–CO_2_ (log*f*O_2_ = −7)	29		8	20	43	
Fefree3	1,160	36	In air (log*f*O_2_ = −0.7)	100					
Fefree4	1,140	36	In air (log*f*O_2_ = −0.7)	100					
Fefree15	1,130	36	In air (log*f*O_2_ = −0.7)	73	1		8	18	
Fefree5	1,100	36	In air (log*f*O_2_ = −0.7)	44		6	15	35	
Fefree7	1,090	36	In air (log*f*O_2_ = −0.7)	43		7	16	34	
Fefree8	1,050	36	In air (log*f*O_2_ = −0.7)	27		7	22	44	
Fefree9	1,000	36	In air (log*f*O_2_ = −0.7)	15		11	23	50	1

P = 1 atm for all experiments (Exp.). Mineral abbreviations: Ol, olivine; Opx, orthopyroxene; Cpx, clinopyroxene; Pl, plagioclase; Ru, rutile.

The liquidus is significantly lower at the more oxygenated condition (log*f*O_2_ = −0.7, in air). At this higher oxygen fugacity, 15 wt.% melt is present at 1,000 °C, coexisting with orthopyroxene + clinopyroxene + plagioclase + rutile. With the temperature increasing, rutile disappears at 1,050 °C (27 wt.% melt), and olivine replaces orthopyroxene at 1,130 °C (73 wt.% melt). The composition is fully molten at 1,140 °C, constraining the liquidus to 1,135 ± 5 °C.

At 1 atm and the intermediate log*f*O_2_ = −7 and 1,150 °C, 29 wt.% of the composition is molten, in equilibrium with the mineral assemblage orthopyroxene + clinopyroxene + plagioclase. At 1,200 °C, the corresponding percentage of melt is 58 wt.%, and the sample is fully molten at 1,230 °C. The liquidus temperature is less well constrained at this *f*O_2_, but, based on the increase in subliquidus melt percentages with increasing temperature, is estimated to be 1,215 ± 15 °C.

The temperature interval between solidus and liquidus, and the minerals coexisting with melt, are similar to observations in an experimental study of the 1-atm melting behavior of natural, iron-bearing terrestrial basalt (51). At a log *f*O_2_ of ∼−11, the solidus of this natural composition is ∼1,080 °C, and the liquidus is ∼1,200 °C, with clinopyroxene, plagioclase, and olivine coexisting with melt until the late disappearance of clinopyroxene just below the liquidus.

In summary, our data indicate that the liquidus of iron-free basalt is lowered by 105 ± 10 °C when log *f*O_2_ is increased by 11 log units. As indicated by dashed lines in Fig. 1, the effect is not linear, and is not restricted to the liquidus. Curves indicating constant melt weight percentages are subparallel to the liquidus temperature trend: Temperatures needed to achieve identical melt weight percentages down to at least 15 remain around 110 °C lower in oxidized conditions than in reduced conditions. Because of our choice of starting material composition, these significant variations in melting behavior cannot be ascribed to variations in cation behavior. In the absence of iron, the only cation that can realistically exhibit multiple valence states would be titanium. However, virtually all Ti in our experiments is expected to be Ti^4+^. Trivalent titanium levels only become significant at conditions more reducing than in our most reduced experiments, and at temperatures much higher than the maximum temperatures achieved here (52). Volatile abundance variations can also be excluded. The maximum C level in our experiments is only ∼0.5 ppm based on its solubility in basaltic silicate melt at 1 atm (53). We conclude that refractory oxygen itself must cause the observed trends.

The abundance of refractory oxygen in silicate magma cannot be measured accurately; its abundance is commonly derived indirectly from measurements of cation abundances, where two oxygens are assigned to each Si atom, one to each Fe^2+^ atom, etc. This may have contributed to the lack of previous observations of the effect we observe. Recently, Fabbrizio et al. (54), in a study of the effect of *f*O_2_ on europium partitioning between silicate minerals and melt, noted that melt percentages increased as a function of increasing *f*O_2_ at constant temperature in their iron-free system, but, as this was not the focus of their study, they did not expand on this observation. We suspect similar observations may have gone unnoticed in other experimental studies. Our results could also shed light on observations of variations in the chemical composition of natural(iron-bearing) mid-ocean ridge basalt (MORB) on Earth as a function of *f*O_2_. Cottrell and Kelley (55) measured a decrease of incompatible trace element abundances with increasing *f*O_2_. This is consistent with increased melt percentages at constant temperature as a function of increasing *f*O_2_.

## Conceptual Model and Implications

Conceptually, the hypothesis that refractory oxygen abundance variations strongly affect melting behavior requires the assumption that oxygen behaves like an incompatible element in systems containing minerals and silicate melt. Increasing its abundance in a bulk composition at high *f*O_2_ conditions leads to a lowering of the activities of the other (cation-bearing) components in silicate melt, leading to enhanced stability of melt with respect to coexisting minerals. We hypothesize that refractory oxygen incorporation into silicate melt occurs through depolymerization of the melt, similar to existing models of melt depolymerization due to the addition of H_2_O (56–60).

In iron-bearing systems, the effects of oxygen fugacity on melting could be at least as large as in the iron-free system. At oxidizing conditions, trivalent iron (which is also an incompatible element) is more abundant than compatible divalent iron, leading to further enhanced melt stability. At reducing conditions, iron metal becomes stable. Metal segregation lowers the iron content of the silicate, which, in itself, is expected to further increase solidus and liquidus temperatures, on top of any increases that can be attributed to oxygen.

**Fig. 2.**
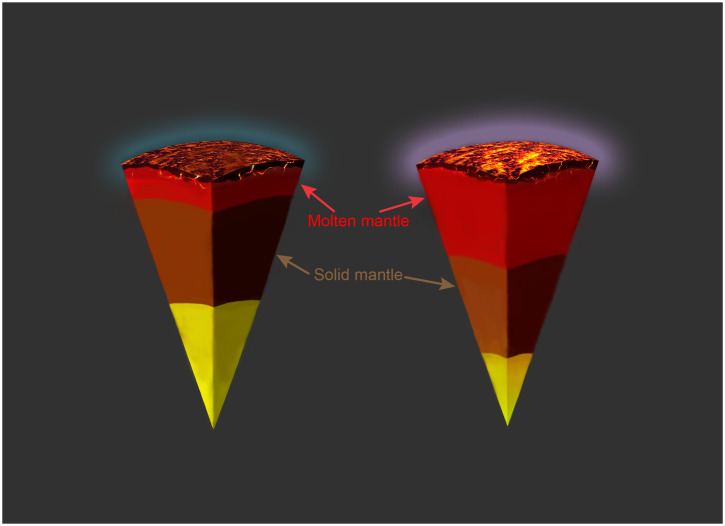
Cartoon illustrating the structure of rocky exoplanets with identical relative cation abundances and thermal profiles at low (Left) and high (Right) planetary oxygen fugacity during their magma ocean stage. Oxidized planets have smaller metal cores (yellow), deeper magma oceans (red), and thicker, more oxidized atmospheres (purple). Solid lower mantles shown in claret.

The cartoon in Fig. 2 illustrates the implications of our finding for the early evolution of rocky exoplanets, by comparing, qualitatively, the structure of two planets with the same relative cation abundances and thermal structure but different refractory oxygen content. In addition to the well-known effect of refractory oxygen content on relative core size (5, 61), our data suggest that higher refractory oxygen abundances cause more extensive melting in the interior. On the one hand, this corresponds to a deeper magma ocean for a given temperature profile, assuming the effect we observe in Fig. 1 down to ∼15 wt.% melt extends down to solidus temperatures and persists at higher pressure. On the other hand, lower liquidus temperatures at higher refractory oxygen abundances imply the magma ocean stage of planetary evolution will extend for longer periods of time. The latter effect may be further strengthened by the fact that deeper magma oceans would lead to more substantial degassing of volatiles from the planetary interior, forming a thicker atmosphere that provides better insulation against planetary cooling. Finally, this thicker atmosphere is likely to be dominated by more-oxidized species given the comparatively high *f*O_2_ of the underlying mantle (47). If the planets contain significant amounts of hydrogen and carbon in their interiors, the main atmospheric constituents produced by outgassing of magma ocean would be H_2_, CO, and CH_4_ at reduced conditions, versus O_2_, H_2_O, and CO_2_ at oxidized conditions.

Our dataset is insufficient at present to develop quantitative models of *f*O_2_-controlled melting and crystallization in exoplanets, but, in principle, with additional experimental data, it should become feasible to better constrain the interior structure of rocky exoplanets based on mass-radius atmospheric composition considerations, including planetary *f*O_2_ as an explicit variable. Because of the link between interior *f*O_2_, magmatism, and extent and duration of planetary outgassing in the magma ocean stage, a coupling with future observations of rocky exoplanet atmospheric compositions and thicknesses may also be achievable.

## Methods

### Starting Materials.

A synthetic Fe-free basalt based on an MORB composition from Melson et al. (50) was used as the starting compositions for our experiments. Following the methods described by Lin et al. (48), all experiments were performed under nominally anhydrous conditions.

The Fe-free basalt starting material was prepared by mixing appropriate proportions of high-purity (99.5 to 99.99%, Alfa Aesar) finely powdered oxides (SiO_2_, TiO_2_, Al_2_O_3_, and MgO) and carbonates (CaCO_3_, Na_2_CO_3_, and K_2_CO_3_). The oxides SiO_2_, TiO_2_, Al_2_O_3_, and MgO were fired overnight at 1,100 °C, and CaCO_3_, Na_2_CO_3_, and K_2_CO_3_ were stored at 110 °C overnight before use. The resulting starting material was homogenized under ethanol in an agate mortar for 1 h, dried in a fume hood, and subsequently decarbonated in an iron-free Pt crucible using a box furnace, by increasing temperature from 650 °C to 1,000 °C over 7 h. The CO_2_-free mixture was then melted for 25 min at 1,550 °C to ensure full chemical homogenization. The melt was quenched rapidly by immersing the bottom of the Pt crucible in water.

Accurate knowledge of the starting material composition enhances the accuracy of mass balance calculations performed subsequently for each experimental run product. To this end, a shard of the starting material glass was mounted in epoxy, ground, polished, and carbon coated for chemical analysis. All remaining glass was crushed into powder in an agate ball mill for 90 min, and then stored at 110 °C for at least 24 h prior to use in the high-temperature experiments.

### The 1-atm High-Temperature Experiments.

Three series of high-temperature, 1-atm experiments on the Fe-free starting composition were designed to run in oxidized (high *f*O_2_) and reduced (low *f*O_2_) conditions. Sample capsules were used instead of wires or foils, in order to maximize sample volumes. A Linn high-temperature box furnace using an ambient air atmosphere was used to achieve oxidized conditions, with temperatures calibrated against noble metal melting points. The oxygen partial pressure of air is assumed to be constant during the experiment, imposing a log*f*O_2_ of −0.7 on the samples.

Two-millimeter outer diameter (OD) Pt capsules with one end welded shut were loaded with compacted starting material. The starting material was first fully melted at 1,200 °C for 30 min, after which the temperature was slowly decreased to the target temperature with a ramp of 5 °C/h to promote crystal growth. Each experiment was then kept at the final target temperature for 36 h to ensure chemical equilibrium between all coexisting phases.

A Gero 1-atm gas-mixing furnace at Vrije Universiteit Amsterdam was used to perform the reduced experiments, using 2-mm OD Pt capsules. The oxygen fugacity of these experiments was controlled using a CO/CO_2_ gas mixture, apportioned according to the *P-T-f*O_2_ table in Deines et al. (62). The oxygen fugacity was verified using an yttria-stabilized ZrO_2_ solid electrolyte sensor. Temperature was monitored with a Pt_70_Rh_30_–Pt_94_Rh_6_ (type B) thermocouple, and calibrated against noble metal melting points before the experiments. The experiment with target temperatures lower than 1,250 °C started by fully melting the charges at 1,250 °C for 30 min, then gradually reducing the temperature to the target temperature at a rate of 5 °C/h. The dwell duration at the target temperature varied between 36 and 40 h (Table 1). The two hottest experiments (1,250 °C and 1,280 °C) were run for 24 h.

### Analytical Techniques.

Experimental run products were mounted in epoxy, polished, and carbon coated for BSE imagery to assess the texture and mineralogy, and for quantitative compositional measurements using a scanning electron microscope (SEM). The chemical composition of the run product phases (minerals and melts) was determined using energy-dispersive spectrometry (EDS) measurements on a JEOL JCM-6000 Secondary Electron Microscope at Vrije Universiteit Amsterdam. Analyses were performed at an accelerating voltage of 15 kV with a focused spot (diameter of <1 μm) and 3-nA beam current, measured by an absorbed current meter attached to the sample stage, for a duration of 30 s. The analyses were performed standardless, with starting material glass and run product analyses performed in the same analytical session. Mineral and melt proportions were determined by mass balance calculations using the EDS data, and are reported in full in *SI Appendix*.

## Supplementary Material

Supplementary File

## Data Availability

All study data are included in the article and *SI Appendix*.

## Change History

October 6, 2022: Figure 1 and its legend have been updated; please see accompanying Correction for details.
