# Supplementary material for: Oxygen controls on magmatism in rocky exoplanets
Source: Proc Natl Acad Sci U S A. 2021 Nov 1;118(45):e2110427118. doi: 10.1073/pnas.2110427118 (PMC8609297; doi:10.1073/pnas.2110427118)
Supplement: Supplementary File [file pnas.2110427118.sapp.pdf]

PNAS
www.pnas.org

Supplementary Information for

Oxygen controls on magmatism in rocky exoplanets

Yanhao Lin[a,1], Wim van Westrenen[a,b], Ho-Kwang Mao[a]

[a]Center for High Pressure Science and Technology Advanced Research, Beijing 100094, People's Republic of China
[b]Department of Earth Sciences, Faculty of Science, Vrije Universiteit Amsterdam, De Boelelaan 1085, 1081 HV Amsterdam, The Netherlands

[1]Corresponding author: Yanhao Lin
Email: yanhao.lin@hpstar.ac.cn

This PDF file includes:

Tables S1

Table S1. Summary of electron microscope analysis results.

Exp.	Conditions				phases	n	SiO_2	TiO_2	Al_2O_3	MgO	CaO	Na_2O	K_2O	Phase proportion (modal %)
	T (°C)	P	Duration (hrs)	LogfO_2										
Starting comp.							55.94	1.59	18.01	9.69	11.99	2.74	0.11	
FefreeIW6	1280	1 atm	24	$CO-CO_2$ (logfO_2 = -11.5)	Glass	5	56.70	1.30	17.93	10.43	12.07	2.06	0.05	100
FefreeIW5	1250		24		Glass	5	56.92	1.47	17.27	10.36	11.72	2.27	0.05	100
FefreeIW7	1230		40		Opx	3	55.38	0.00	1.94	38.63	3.99	-	0.07	1
					Glass	5	56.18	1.37	18.17	9.71	12.62	2.49	0.35	99
FefreeIW4	1200		36		Opx	4	57.50	0.15	0.47	38.96	2.92	-	0.10	8
					Cpx	4	51.88	1.50	6.66	22.01	17.95	-	0.05	16
					Pl	4	53.59	0.27	28.22	0.97	13.58	3.36	-	40
					Glass	5	60.73	3.20	15.95	7.78	9.73	2.63	0.40	36
FefreeIW2	1150		36		Opx	4	58.26	0.81	0.43	38.03	2.89	-	-	8
					Cpx	5	51.44	1.43	6.83	22.55	17.75	-	-	27
					Pl	4	54.24	0.27	29.25	0.55	12.53	3.70	-	46.5
					Ru	3	-	100	-	-	-	-	-	0.5
					Glass	5	68.58	4.89	14.36	3.74	6.22	1.67	0.57	18
FefreeIW1	1100		36		Cpx	4	51.94	1.54	5.85	22.37	18.31	-	-	30
					Pl	5	55.99	0.87	24.29	3.18	12.77	3.57	-	56
					Ru	3	-	100	-	-	-	-	-	1
					Glass	5	74.11	2.30	15.67	1.15	5.63	1.84	0.59	13
Fefree-7_3	1230		40	$CO-CO_2$ (logfO_2 = -7)	Glass	5	55.06	1.41	19.20	9.02	12.51	2.56	0.22	100
Fefree-7_1	1200		36		Opx	4	55.52	-	2.43	37.71	4.31	-	-	6
					Cpx	4	52.71	1.29	3.12	23.72	19.16	-	-	15
					Pl	4	50.83	0.31	31.01	0.61	14.63	2.94	-	21
					Glass	5	58.65	2.50	18.81	6.52	9.99	3.54	-	58
Fefree-7_2	1150		36		Opx	3	56.38	0.62	1.29	37.95	3.77	-	-	8
					Cpx	4	52.81	1.48	2.89	24.14	18.67	-	-	20
					Pl	4	51.73	0.09	30.33	0.54	13.72	3.59	-	43
					Glass	4	65.81	3.94	15.55	5.21	7.23	2.61	0.24	29
Fefree3	1160		36	In air (logfO_2 = -0.7)	Glass	5	55.88	1.45	18.19	9.53	12.07	2.77	0.10	100
Fefree4	1140		36		Glass	9	56.16	1.66	17.57	10.00	11.96	2.64	0.11	100
Fefree15	1130		36		Ol	3	39.50	0.04	-	60.40	-	-	-	1
					Cpx	5	53.11	0.44	2.71	25.10	18.65	-	-	8
					Pl	5	52.11	0.70	31.30	0.41	13.16	3.44	-	18
					Glass	5	57.48	1.80	16.80	10.71	10.81	2.71	0.10	73
Fefree5	1100		36		Opx	4	56.20	0.48	1.00	40.44	3.04	-	-	6
					Cpx	6	52.75	1.19	2.86	24.20	19.01	-	-	15
					Pl	5	52.50	0.26	30.30	0.26	13.51	3.60	-	35
					Glass	5	60.29	2.97	16.19	7.93	9.81	2.81	-	44
Fefree7	1090		36		Opx	4	55.88	0.42	0.71	39.12	3.99	-	-	7
					Cpx	4	52.44	1.45	3.04	24.37	18.69	-	-	16
					Pl	3	51.80	0.41	30.91	0.11	14.01	3.30	-	34
					Glass	7	60.78	3.06	16.10	7.32	9.44	3.19	-	43
Fefree8	1050		36		Opx	5	57.41	0.94	1.99	37.63	3.42	-	-	7
					Cpx	3	51.31	1.46	4.95	23.79	18.49	-	-	22
					Pl	7	53.16	0.22	29.41	0.98	13.20	3.72	-	44
					Glass	7	64.22	4.37	14.91	6.28	7.45	2.25	0.55	27
Fefree9	1000		36		Opx	3	57.13	0.57	0.21	38.44	3.87	-	-	11
					Cpx	3	52.15	1.51	3.72	23.90	18.72	-	-	23
					Pl	3	51.85	0.18	30.61	0.21	13.99	3.55	-	50
					Ru	3	-	100	-	-	-	-	-	1
					Glass	6	79.84	2.25	12.60	0.81	2.21	1.34	0.96	15

T, temperature; P, pressure; n, number of analyses; Duration here reprensents the time stable at the aim temperature;
Mineral abbreviations: Ol, olivine; Opx, orthopyroxene; Cpx, clinopyroxene; Pl, plagioclase; Ru, rutile;
Phase proportion calculated using least squares mass balance, and compositions from SEM in wt. % oxides;
Standard deviations based on multiple analyses for each phase is less than 2%; ' - ' means the vaule lowers than the detection limit.
